# Preventive Behaviors of French Cancer Patients and How They Changed During the COVID-19 Outbreak (PAPESCO-19 Study)

**DOI:** 10.3389/ijph.2025.1608450

**Published:** 2025-12-02

**Authors:** Pauline Petit, Myriam Blanchin, Frederic Bigot, Hakim Mahammedi, Mario Campone, Frederique Penault-Llorca, Ke Zhou, Valerie Seegers, Martine Marie Bellanger, Audrey Blanc-Lapierre

**Affiliations:** 1 Department of Biostatistics, Institut de Cancérologie de l’Ouest (ICO), Saint-Herblain, France; 2 Nantes Université, Université de Tours, Institut national de la santé et de la recherche médicale (INSERM), Methods in Patients-Centered Outcomes and Health Research, SPHERE, Nantes, France; 3 Department of Medical Oncology, ICO, Angers, France; 4 Department of Medical Oncology, Centre Jean Perrin, Clermont-Ferrand, Auvergne, France; 5 INSERM U1240 Imagerie Moléculaire et Stratégies Théranostiques (IMOST), Clermont-Ferrand, Auvergne, France; 6 Department of Biopathology and INSERM U1240, Centre Jean Perrin, Clermont-Ferrand, Auvergne, France; 7 Department of Human and Social Sciences, ICO, Saint-Herblain, France; 8 Department of Social Sciences, EHESP School of Public Health, Rennes, France

**Keywords:** COVID-19, cancer patients, preventive behavior, latent classes, trajectories

## Abstract

**Objectives:**

In the context of the COVID-19 epidemic, adopting preventive behavior could be defined as complying with recommendations issued by public health authorities. The aim of this study was to investigate heterogeneity of preventive behavior changes over time among cancer patients (CPs) during outbreak.

**Methods:**

The PAPESCO-19 study is a multicenter prospective cohort including 893 CPs from French comprehensive cancer centers (June 2020- June 2021). During the 1-year follow-up, CPs completed questionnaires on socio-demographics, lifestyle and COVID-19-related history. Biological and clinical data were collected from medical records. We used the R package lcmm to determine the different classes of preventive behavior trajectories in CPs.

**Results:**

Between June 2020 and April 2022, over two-thirds of CPs reported wearing a mask during all outings. Only one class of preventive behavior was identified. Female CPs, those on sick leave, CPs unable to work due to health reasons and those spending most of the day at home, showed more preventive behavior. CPs with two or more children were less likely to adopt preventive behavior.

**Conclusion:**

No patient clinical characteristics were associated with preventive behavior.

## Introduction

Coronavirus disease 2019 (COVID-19), caused by severe acute respiratory syndrome coronavirus 2 (SARS-CoV-2), is an infectious disease presenting symptoms ranging from fever, cough and headaches to severe respiratory issues. The first cases in France were detected in January 2020 [1]. To limit the spread of the virus, three lockdowns were set: from 17 March 2020 to 11 May 2020, from 30 October 2020 to 15 December 2020 and from 3 April 2021 to 3 May 2021. During these lockdowns, so-called “non-essential” shops and retail stores, and places frequented by the public were closed and a stay home policy was enforced except for a limited number of reasons. Between these lockdowns, curfews were also introduced. Furthermore, the COVID-19 vaccination campaign started on 27 December 2020.

The government set a series of public health and social measures, encompassing precautions such as mask-wearing, regular ventilation, hand hygiene, social distancing, and coughing and sneezing into the bend of the elbow. The aim of these measures was to encourage the adoption of preventive behavior. Preventive health behavior has been defined as “any activity undertaken by an individual who believes himself to be healthy for the purpose of preventing or detecting illness in an asymptomatic state” [2]. In the COVID-19 context, the adoption of preventive health behavior could be defined as adherence to the main behavioral recommendations issued by public health authorities. However, our previous research did not enable us to identify which specific barrier gesture was more indicative of preventive health behavior. A French national survey (CoviPrev) which regularly assessed indicators for adopting preventive measures throughout the COVID-19 epidemic, showed changes over time. The systematic adoption of mask wearing showed variability according to demographic and socioeconomic factors, including gender, age, occupation, employment status, perceived financial situation, health literacy, the presence of others in the household, the presence of a child under 16 years, a history of psychological disorders, and a potential risk of developing a severe form of COVID-19 [3].

From the beginning of the outbreak, cancer patients (CPs) were suspected of being at risk of severe COVID (SARS-CoV-2) infection. Further evidence demonstrated that CPs represented a high mortality risk group (RR = 1.7), especially younger ones, patients who had lung or hematologic cancers, or those treated with chemotherapy [4]. By reducing the risk of contamination, adopting preventive measures against COVID-19 infection, contributed to reduce the risk that CPs developed serious forms of COVID-19 and of cancer treatment being suspended. The literature on the preventive behaviors of CPs during the epidemic is limited, mostly being cross-sectional studies. Among cancer patients in the Middle East and North Africa, male gender, divorced or widowed marital status, disease in remission, the cancellation of a medical appointment or treatment session, and a lack of knowledge about COVID-19 were associated with poor precautionary practices [5].

Among adolescents and young adults with cancer in Canada, adherence to precautionary behaviors related to COVID-19 was lower in males than in females [6].

Given the data available in the general French population or on cancer patients, we assume that the preventive behavior of CPs may have changed during the epidemic, and that different factors may have been associated with the adoption of preventive behavior in this population. Furthermore, CPs do not share the same clinical, sociodemographic and lifestyle characteristics. It could thus be expected that different trajectories of preventive behaviors would be observed in the CPs according to their individual characteristics.

### Objectives

The PAPESCO-19 (PAtients et PErsonnels de Santé des Centres de Lutte Contre le Cancer pendant l’épidémie de COVID-19) study investigated the impact of COVID-19 on CPs and healthcare workers in French Comprehensive Cancer Centers.

The present study had a threefold objective: to define and model the preventive behavior, to study its change over time, and to determine the different profiles of behavioral change in CPs over the 2-year period of the PAPESCO-19 cohort. Examining the variables associated with these profiles will help identify potential levers for enhancing preventive behavior during epidemic waves in such populations. In this study, “preventive behavior” was defined as adherence to the main practices recommended by public health authorities to limit COVID-19 transmission — namely, hand washing, mask wearing, physical distancing, and limitation of outings. It is distinguished from risk perception and structural determinants (contextual, material, or institutional factors).

## Methods

### Study Design and Setting

The PAPESCO-19 prospective multicenter cohort study took place in four French comprehensive cancer centers located in Clermont-Ferrand, Nancy, Angers and Nantes (the study has been described previously [7, 8]). Adult patients attending the centers for active treatment or monitoring of solid cancers were recruited between 17 June 2020 and 16 June 2021. Participants were followed up every 3 months over a full year (last follow-up completed on 28 June 2022). At baseline and follow-up, each participant completed self-report questionnaires online on socio-demographics, lifestyle and COVID-19 related history. Clinical research associates collected baseline demographic (age and sex), biological, and clinical data from their medical records.

Initial analyses showed that most CPs (70%) were vaccinated during the follow-up period [7] and that 8% of CPs had experienced at least one SARS-CoV-2 positive serological test or RT-PCR result [8]. All participants signed an informed consent form, and the study was conducted in accordance with the Declaration of Helsinki. The Ethics Committee (CPP-IDF VIII, Boulogne-Billancourt) approved the study (number 20.04.15) on 15 May 2020. This study was registered at ClinicalTrials.gov, Identifier: NCT04421625.

### Data Collection

Patients provided information on their occupational categories and on their living conditions (housing, household composition) at baseline. Work status and conditions (teleworking, on-site work, sick leave, unemployment), frequency of weekly trips, adoption of barrier measures, history of a COVID-19 infection confirmed by a PCR, antigenic or saliva test and COVID-19 vaccination (dates of vaccine injections, intention to get vaccinated) were reported quarterly.

### Variables

The inclusion period lasted a year encompassing three waves of the epidemic, two national lockdowns and two periods of partial curfews. The time of each patient’s first visit (time of study entry) was thus not relevant. Hence, the date of the first patient inclusion was considered the reference time (T0) for everyone to account for possible changes in definition and experience of preventive behavior during the inclusion period. The time variable of interest for the analyses was defined as the time elapsed between the first patient inclusion and the date of questionnaire completion, expressed in weeks.

Variables that could define or influence patients’ preventive behavior (Supplementary Figure SB2) were selected by excluding categorical variables when either the distribution of response categories did not vary over time, or one category was overrepresented (percentage >95%), or variables were suspected of being redundant due to a high correlation (0.8).

### Statistical Analysis

Patients from the Nantes, Angers or Clermont-Ferrand centers who completed at least one questionnaire were included in the analyses.

#### Descriptive Analysis

Categorical distribution of variables defining preventive behavior was illustrated with a bar diagram according to the time elapsed since T0 split into 2-month intervals from 15 June 2020 until the last questionnaire was submitted. Missing data rates were low, and no imputation was performed.

#### Definition and Modelling of the Preventive Behavior

Preventive behavior is not directly observable and was hence represented by a latent variable. However, preventive behavior has been defined by the variables previously classified as defining preventive behavior and that were observed. It was assumed that the level of preventive behavior was reflected in the observed answers on the definition variables. In fact, for a high level of preventive behavior, we could expect very frequent hand washing for example.

Alternatively, it would have been possible to sum the answers to definition variable in the aptly named sum score. Yet, different response profiles could result in the same score making interpretation of definition variables poorer. Moreover, sum scores do not use all available information as the HCP score cannot be computed if an answer to definition variables is missing for the patient. Hence, a latent variable approach was favored to ensure use of all available information and to avoid masking relevant information from definition variables.

A graded-response model (GRM) [9, 10], a cumulative probit model from the item response theory, was used to link the preventive behavior to the observed definition variables of a binary or categorical nature. The preventive behavior is represented by the latent variable, continuous by essence and characterized by its distribution parameters (mean and variance). The higher the latent variable, the less preventive the behavior. In this regression model, the probability to answer one of the answer categories is explained by the latent variable level (preventive behavior) and characteristics of definition variables (discrimination and location). Definition variables can be more or less important in the definition of preventive behavior. The importance or the strength of association with preventive behavior is expressed with the so-called discrimination parameter for each observed variable. The higher the discrimination parameter of a definition variable, the higher its importance in the definition of preventive behavior. Some preventive measures might be easier to adopt than others resulting in a lower required preventive behavior to answer favorably on these variables. The different location parameters of definition variables indicate the levels of preventive behavior that needed to be reached to endorse each response category.

#### Change of Preventive Behavior and Heterogeneity of the Trajectories

As preventive behavior was expected to change over time, the latent variable was considered as a continuous latent process. Mixed models are the preferred models to estimate the trajectory of a continuous outcome over time. In this study, different trajectories were expected as the CPs is a heterogeneous population. Hence, latent class mixed models (LCMM), an extension of linear mixed models suited to heterogeneous subpopulation analysis, were combined with the GRM to identify different profiles of preventive behavior. Instead of modelling a unique trajectory for all the sample, LCMM estimate a specific mean trajectory of preventive behavior in each subpopulation (a latent class) defined by an unobserved grouping variable [11]. Each class-specific mean trajectory was defined using a linear mixed model with the latent process defined above as the dependent variable. Latent class analysis does not directly assign individuals to latent classes during estimation but rather the probabilities of belonging to each class are generated for each individual using a multinomial logistic model.

#### Modelling Strategy

The best fitting shape of the mean trajectory of preventive behavior over time between polynomials (quadratic, cubic) or quantile splines (3, 4 or 5 knots) was determined by comparing the BIC of five one-class LCMM. As the grouping variable was latent, the number of latent classes was not known prior to the analysis. Latent class mixed models were thus estimated with 1–5 latent classes to highlight some overall differences in the changes in CPs’ preventive behavior, and to avoid pointing out very specific subgroups. The optimal number of classes was based on the BIC, the size of the minimal latent class (10% or 15% depending on the number of classes), and the interpretability of the latent classes.

In case of a single-class optimal model, the LCMM was re-estimated with adjustment for center, sex, age and cancer location. Either a set of sociodemographic variables or a set of clinical variables, living conditions and self-reported COVID-19 related information was investigated in a backward selection procedure to identify the factors significantly (p < 0.10) associated with the preventive behavior. Eventually, the mean predicted trajectories of preventive behavior in each class and the location parameters were plotted. Further information about the statistical analysis method is provided in the electronic Supplementary Material.

The Ennov Clinical system was used for data collection and R, version 4.2.2, was used for statistical analysis. The analysis combining latent class mixed model and GRM was performed with the multlcmm function in the R package lcmm [12].

## Results

### Participants

Of the 893 cancer patients included in the study, 864 met the inclusion criteria and completed at least one questionnaire (Supplementary Figure SB1). Of them, 480 CPs completed the 1-year follow-up visit (including those with missed intermediate visits or incomplete data), 113 deceased and 271 dropped out. Differences in center and cancer stage were observed between participants who dropped out and those who completed the study or were followed until death (Supplementary Table SB1).

### Variable Selection

Among the variables from the PAPESCO questionnaires that could either influence or define the preventive behavior (Supplementary Figure SB2), hand washing, mask wearing, physical distance and number of outings were assumed to define preventive behavior. The variable “self-reported vaccination status” was not considered to define preventive behavior as vaccination may not have represented an intentional choice of the patient. It may instead have influenced the preventive behavior. In addition, the variable “self-reported intention to get vaccinated” was also not considered as it was only relevant during the limited period between the introduction of vaccination and its widespread uptake.

### Clinical and Sociodemographic Characteristics

Although inclusions began later in Clermont-Ferrand (Supplementary Figure SB3), this center included more patients than Nantes and Angers (Table 1). The cohort of CPs consisted mainly of women (68%). About half were aged under 65 (56%). Almost all the patients (97%) were undergoing treatment at inclusion. The most frequent cancer was breast cancer (44%), and the most frequent cancer stage was metastatic (52%). Most patients had at least one comorbidity (64%), were non-smokers (60%) and were of normal weight (46%) or were overweight (33%). At inclusion, half of the CPs reported that they belonged to intermediate professions (25%) or were employees (26%), and more than half reported being retired or without professional activity (56%). Most patients lived with at least one other person (79%), stayed at home most of the day (82%) and did not live with children under 18 (83%). Very few COVID-19 infections were reported, in fact only 23% of patients (7 patients) reported having been infected with COVID-19 in the previous 3 months during the first questionnaire period (06/15/2020 – 06/14/2020, results not shown). This variable was widely affected by missing data. Almost all CPs reported having been vaccinated in the last year of the study.

**TABLE 1:** Reported clinical characteristics and sociodemographic data for cancers patients in the PAPESCO (PAtients et PErsonnels de Santé des Centres de Lutte Contre le Cancer pendant l’épidémie de COVID-19) study (France 2020–2022) at the inclusion visit (N = 864).

Characteristic	n (%)
Center	
Nantes	263 (30%)
Angers	259 (30%)
Clermont-ferrand	342 (40%)
Sex	
Man	277 (32%)
Woman	587 (68%)
Age	
Under 65 years old	480 (56%)
65 years and older	384 (44%)
Treatment status	
Patients in follow-up	26 (3%)
Patients in treatment	838 (97%)
Cancer location	
Prostate	56 (7%)
Breast	377 (44%)
Digestive	57 (7%)
Lung	69 (8%)
Uterine, cervical or endometrial	100 (12%)
Urological	66 (8%)
Other	130 (15%)
Missing	9
Cancer stage	
Localized	239 (29%)
Locally advanced	162 (19%)
Metastatic	437 (52%)
Missing	26
One or more comorbidities^a^	554 (64%)
Missing	1
Tobacco smoking status	
Non-smoker	519 (60%)
Former smoker	245 (28%)
Current smoker	100 (12%)
Body mass index classifications	
Underweight	40 (5%)
Normal weight	353 (46%)
Overweight	256 (33%)
Obesity	125 (16%)
Missing	90
Socio-professional category	
Farmers	20 (3%)
Craftsmen, merchants, company managers	62 (8%)
Executives and higher intellectual professions	128 (16%)
Intermediate professions	197 (25%)
Employees	201 (26%)
Workers	82 (10%)
Never worked	13 (2%)
Other	78 (10%)
Missing	83
Employment situation	
Working or studying	88 (11%)
Sick leave or not working for health reasons	265 (33%)
Retired or without professional activity	450 (56%)
Missing	61
Spending most of the day at home	
No, unrestricted	111 (14%)
No, must go out to work	37 (45%)
Yes	650 (81%)
Missing	66
House location	
City, more than 100,000 residents	119 (15%)
City, 20,000 to 100,000 residents	96 (12%)
City, less than 20,000 residents	193 (24%)
Rural area	401 (50%)
Missing	55
Living with at least one other person	664 (79%)
Missing	24
Number of children or grandchildren under the age of 18 at home	
No children	687 (83%)
One child	68 (8%)
Two children or more	76 (9%)
Missing	33

^a^ Comorbidities included hypertension, diabetes, chronic respiratory failure, chronic kidney failure, heart failure, weight loss, autoimmune disease, surgery under general anesthesia in the previous 12 months. They were selected among risk factors for severe forms of COVID-19.

### Variables Used to Define Preventive Behavior

**FIGURE 1:**
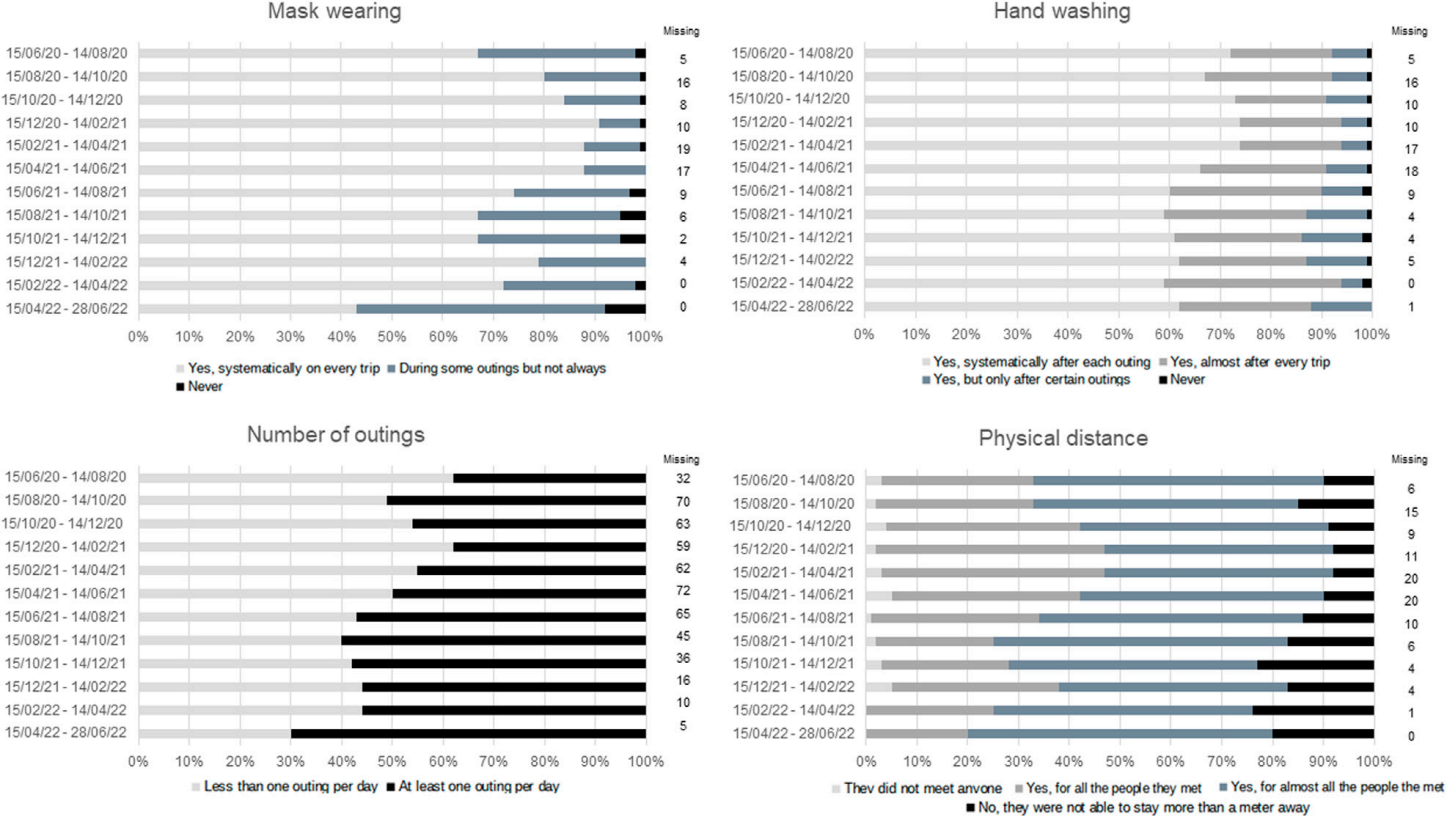
Bar diagram of variables defining preventive behavior (PAPESCO: PAtients et PErsonnel de Santé des Centres de Lutte Contre le Cancer pendant la pandémie de Covid-19, France 2020–2022). The distribution (in percentage of patients) of categorical variables defining preventive behaviors (number of outings during the week, mask wearing, physical distancing and hand washing) is presented in 2-month intervals.

Figure 1 shows the distribution of variables defining preventive behavior (number of outings during the week, mask wearing, physical distancing and hand washing) in 2-month time intervals. Mask wearing and hand washing were reported to be the most followed protective measures. Most patients (62%) limited themselves to less than one outing per day during the period following the first lockdown (March–May 2020).

### Trajectories of Preventive Behavior

According to the BIC criterion, the one-class LCMM provides the best fit when the mean trajectory of preventive behavior was modelled with a 5-knot quantile spline (results not shown).

**FIGURE 2:**
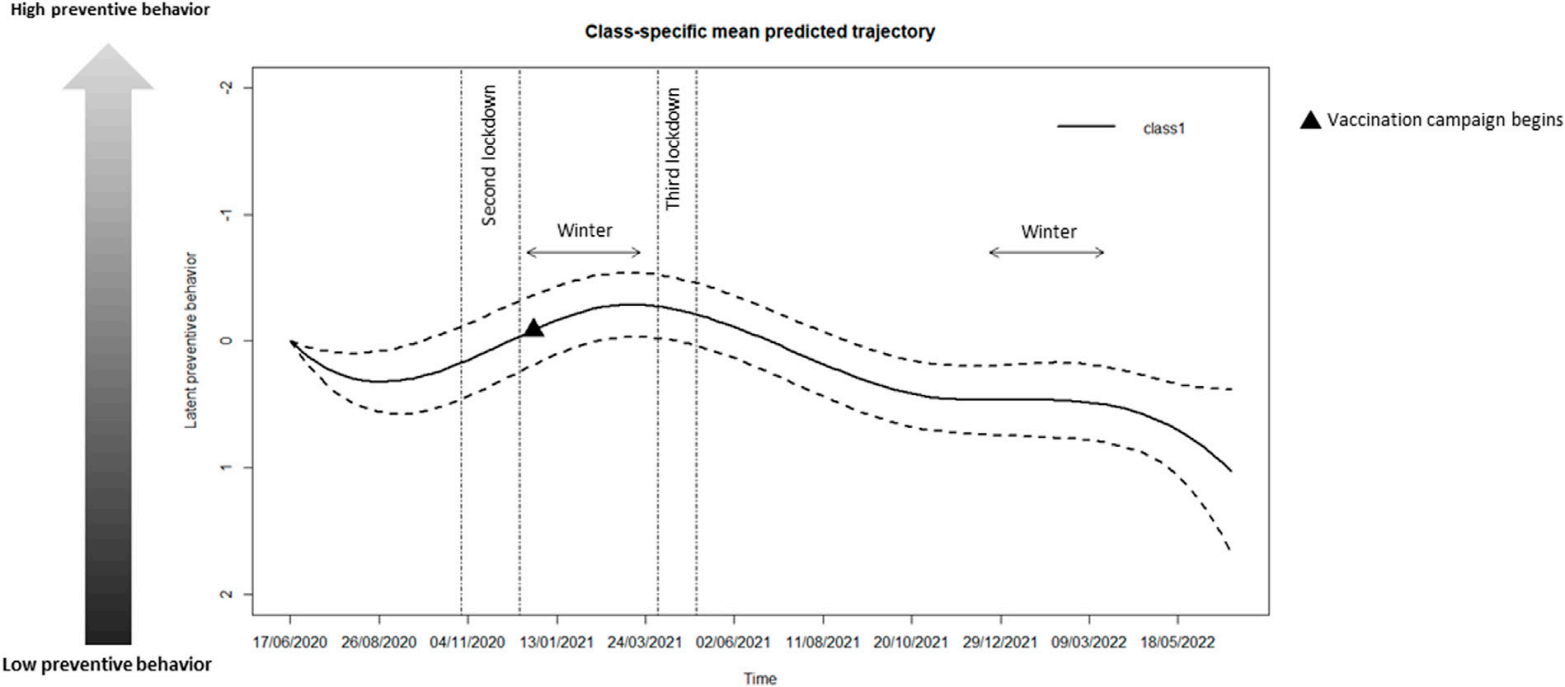
Mean predicted trajectory of preventive behavior (PAPESCO: PAtients et PErsonnel de Santé des Centres de Lutte Contre le Cancer pendant la pandémie de Covid-19, France, 2020–2022). This figure shows the mean trajectory (with corresponding confidence intervals) of preventive behavior estimated over the study period using a latent class mixed model, as well as national lockdowns.

Among the models with a number of latent classes varying from 1 to 5, LCMM with 3 or 4 latent classes did not achieve convergence. The one-class model was selected among the convergent models. The preventive behavior trajectory seems to be homogeneous among the CPs in our data. Hence, a unique trajectory of preventive behavior was estimated, assumed to be on average the same for all CPs. The estimated mean trajectory and confidence intervals over the study period are represented in Figure 2 as well as national lockdowns. Cancer patients seemed to adopt less preventive behavior after the first lockdown and the end of the first peak of cases as the mean of preventive behavior decreased from June 2020 to August 2020 simultaneously with ease of restrictions. On the contrary, the preventive behavior mean increased from September 2020 as the cases began to rise again. The mean continued to increase until the end of Winter 2020–2021 as France entered in the second COVID wave with announcement of curfews in mid-October and a second nationwide lockdown from November 2020 to mid-December 2020 (schools remained opened) followed by more or less strict curfews. Consequently, cancer patients seemed to adopt a more and more preventive behavior during the second lockdown and winter 2020–2021 During this period, mask wearing became mandatory at the beginning of November 2020. After the start of the vaccination campaign at the end of December 2020, with high-priority access for cancer patients under treatment from mid-January 2021, an inflection in the curve was observed. From the third lockdown (schools closed) onwards corresponding to the third epidemic wave until fall 2021, it seems that CPs behavior was less and less preventive while curfews were imposed one after another until mid-June 2021, a health pass to access public venues such as restaurants, theatres and cinemas was imposed in late July 2021 and incidence rose again during summer 2021. Despite a peak in COVID-19 incidence in December 2021 [13], their behavior did not change until spring 2022. From May 2022 onwards, patients reported even less preventive behavior, corresponding to the end of the main barrier measures.

### Factors That May Have Impacted Preventive Behavior

After adjustment for center, sex, age and cancer location, self-reported variables pertaining to employment situation, spending most of the day living at home and number of children or grandchildren under the age of 18 at home were significantly associated with preventive behavior in the final model (Table 2). In fact, women seemed to have a lower mean level of latent variables throughout the study period, indicating higher preventive behavior. In addition, patients being off sick or not working for health reasons adopted more preventive behavior on average than patients working when entering the study. Patients spending most of the day at home adopted more preventive behavior than patients not spending most of the day at home. Finally, patients living with two or more children appeared to adopt less preventive behavior than patients living with no children. However, center, cancer location and age did not appear to be significantly associated with preventive behavior.

**TABLE 2:** Estimated parameters in the final multivariate latent class mixed model (PAPESCO: PAtients et PErsonnels de Santé des Centres de Lutte Contre le Cancer pendant l’épidémie de COVID-19, France 2020–2022).

Parameters	Estimation^a^	Standard error	P-value
Intercept	0.000	-	-
1st spline knots	−0.245	0.809	0.762
2nd spline knots	1.342	0.607	0.027
3rd spline knots	−2.656	1.155	0.021
4th spline knots	0.688	0.984	0.484
5th spline knots	−0.536	1.083	0.621
Center			
Nantes	Reference		
Angers	−0.390	0.242	0.106
Clermont-ferrand	0.415	0.257	0.107
Sex			
Man	Reference		
Woman	−1.055	0.371	0.004
Age			
Under 65 years old	Reference		
65 years and older	−0.424	0.267	0.112
Cancer location			
Prostate	Reference		
Breast	0.183	0.493	0.711
Digestive	−0.237	0.483	0.624
Lung	−0.476	0.488	0.329
Uterine, cervical or endometrial	0.074	0.545	0.891
Urological	−0.284	0.453	0.531
Other	−0.140	0.447	0.754
Employment situation			
Working or studying	Reference		
Sick leave or not working for health reasons	−0.800	0.372	0.031
Retired or without professional activity	−0.669	0.413	0.106
Spending most of the day at home			
Yes	Reference		
No, must go out to work	1.239	0.564	0.028
No, unrestricted	1.543	0.403	<0.001
Number of children or grandchildren under the age of 18			
No children	Reference		
Two children or more	0.742	0.329	0.024
One child	0.001	0.117	0.989
Random effects			
Variance (intercept)	1.000	-	-
Variance (slope)	5.10^–5^	1.10^–4^	-
Covariance (intercept, slope)	0.007	0.010	0.49

^a^ Factors with positive coefficients are associated with less preventive behavior.

**FIGURE 3:**
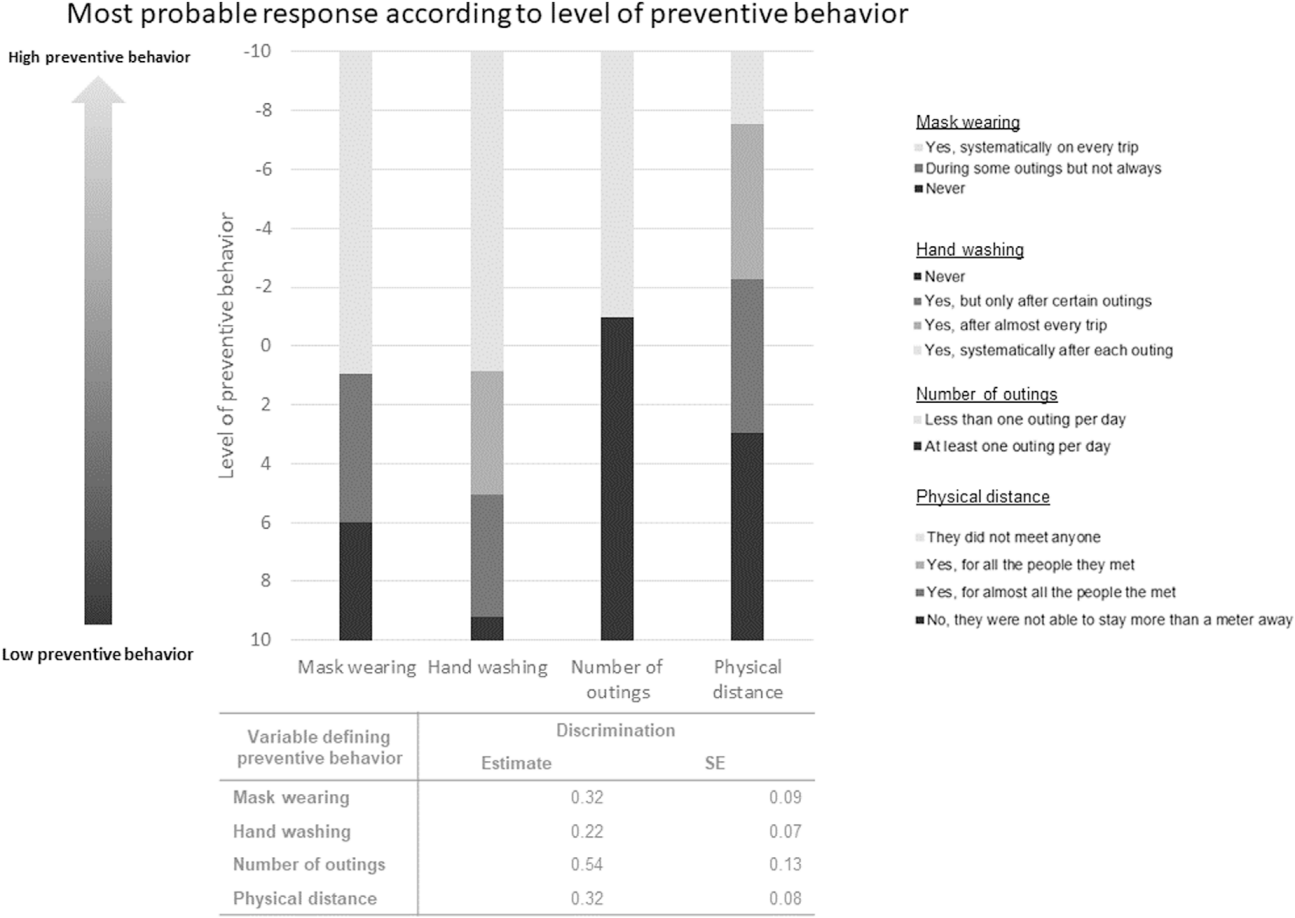
Most probable response category according to the level of preventive behavior (PAPESCO: PAtients et PErsonnel de Santé des Centres de Lutte Contre le Cancer pendant la pandémie de Covid-19, France 2020–2022). This figure represents the most probable response category for each definition variable based on the level of latent variable of preventive behavior estimated in the latent class mixed model. The thresholds between adjacent response categories are given by the estimated values for the location parameters.

Figure 3 shows the most probable response category for each definition variable depending on the level of preventive behavior. Thresholds between each response category are given by estimated values for location parameters. Physical distancing had the lowest location meaning that a very high level of preventive behavior was required to respond to the most favorable answer categories (“Yes, for all the people they met” and “They did not meet anyone”) in the physical distancing variable. Hence, physical distancing seems the most difficult preventive measure to adopt. On the contrary, hand-washing had the highest location indicating that even CPs with low preventive behavior easily answer a more favorable response than “never”. Consequently, we can consider that hand washing is the easiest preventive measure to adopt. Similarly, response profiles of preventive measures adoption can be drawn from Figure 3. The most probable response profile for CPs with a low level of preventive behavior, e.g., 8 on Figure 3, was never wearing mask, go out at least once a day, not able to maintain physical distance and occasional handwashing. On the contrary, patients with a high level of preventive behavior (e.g., −6) have most probably answered that they always wore a mask and washed their hands, went out less than once a day and often maintained physical distance. The most and least discriminating variables were the number of outings and hand washing making them the most and least important variables respectively in the definition of preventive behavior (Figure 3).

## Discussion

### Main Findings

The study had a threefold objective: to define and model preventive behavior, to study its change over time, and to determine the different profiles of behavioral change. We identified a single behavior change profile, suggesting that our CP population was homogeneous in terms of preventive behavior during the COVID-19 epidemic. Patients were required to attend healthcare facilities regularly, where mask wearing had been mandatory since June 2020. They were therefore particularly aware of the importance of adhering to this preventive measure, as illustrated in Figure 1. Moreover, they were aware of their increased risk of developing severe COVID-19. Preventive behavior increased during winter 2020/2021, whereas lockdowns did not seem to modify behavior. This could be explained by the high proportion of patients not working. Female CPs and CPs off sick or not working for health reasons appeared to adopt more preventive behavior than male CPs and working CPs, respectively. While CPs living with two children or more and CPs not spending most of time at home adopted less preventive behavior than patients living without children and CPs spending most of the day at home. Unexpectedly, smoking status, age and cancer location were not associated with preventive behavior. The lack of associations with clinical characteristics may be due to the estimation of a unique trajectory of preventive behavior. Regarding smoking, the small proportion of current smokers (12%) is probably involved. Some cancer-related clinical characteristics may also have been indirectly captured by other variables, such as employment status.

### Strengths and Limitations

The CP population was a very under-studied population in terms of their preventive behavior during the COVID-19 epidemic. Our study provides insights into the population of patients attending cancer centers in France during the 2 years following the first national lockdown. However, the PAPESCO-19 study may suffer from some selection bias. First, the participating centers represent only 2 different regions (2 centers are in the same region). Besides, recruitment was carried out by the center’s physicians, and we suspect, for example, that inclusions were mainly made by medical oncologists and very few by radiotherapists. Furthermore, it is likely that patients that agreed to participate were the most conscientious and were thus more prone to adopt preventive behavior, which could explain the homogeneity of the population. The PAPESCO study population may thus not be representative of cancer patients in France. In addition, smoking status, for example, is a risk factor for many cancers and is expected to be associated with poor preventive health behavior but was not very prevalent in patients included in the study. The overrepresentation of women and breast cancer cases among PAPESCO participants from Nantes and Angers may have contributed to the identification of a single preventive behavior profile. We might expect different profiles of preventive behavior against COVID-19 in more heterogeneous samples, including a more important part of cancers attributable to lifestyle behaviors for example. It should be noted, however, that cancer site was not found to be associated with the mean trajectory of preventive behavior over time in our analyses. In addition, adjustment for gender—a factor influencing both study participation and preventive behavior—contributes to reduce the impact of selection bias on the estimates obtained for the other explanatory variables.

The apparent uniformity in patients’ preventive behaviors may partly stem from methodological constraints, such as the use of variables that do not sufficiently discriminate between different preventive behaviors, as well as limited statistical power.

Despite the aforementioned limitations, our study presents a major strength through its longitudinal design and the one-step approach adopted to model preventive behavior.

### Comparison of Findings With Those Reported in the Literature

Studies of preventive behavior in the context of the COVID-19 pandemic are mainly cross-sectional [5, 6, 14, 15]. Therefore, we could not compare the CP trajectory of preventive behavior during the COVID-19 epidemic with the general population or with a period outside the COVID-19 setting as no suitable data were available. Nevertheless, it is possible to compare some of our results with data from population-based cross-sectional studies.

In France, the CoviPrev study evaluated the adoption of wearing masks in public and the relative frequency of hand washing in waves of representative samples from the general population [3]. From their results, the relative frequency of systematic mask wearing, and systematic hand washing were comparable to those of our CP population. They observed the same trend for increased mask wearing in winter 2020/2021. However, their study did not make it possible to track behavioral changes in the same individuals because the samples differed from one survey wave to the next.

Cross-sectional comparison of COVID-19 preventive behavior in CPs and in the general population was performed through 4 online surveys in Avril-June [14], June [15, 16] or August/September 2020 [17]. In three of these surveys [14, 16, 17], participants with cancer were more likely to report engaging in preventive behaviors, such as social distancing.

Regarding factors associated with preventive behavior against COVID-19, women have been found to be keener to adopt preventive hygiene and social distancing behaviors in web surveys [18, 19].

In the SAPRIS [20] and EPICOV studies [21] estimating the seroprevalence of SARS-CoV-2 infection in the French adult population at the end of the first lockdown, individuals living with children were more likely to be infected with COVID-19. It is difficult, however, to determine to what extent this can be attributed to less preventive behavior or to greater exposure to the virus brought into the household by children. Findings from the French CoviPrev repeated cross-sectional surveys indicate that gender, employment status, and family composition were also associated with mask wearing. The absence of preventive behavior patterns specific to cancer patients is a noteworthy finding, suggesting that general public health recommendations may be applicable to this population without the need to target a particular risk factor for suboptimal preventive behavior.

### Implications for Public Health and Perspectives

Improved characterization of preventive behavior in cancer patients will facilitate more adequate interpretation of data regarding vaccination campaign effectiveness, contamination risk factors, or the incidence of viral respiratory infection in this population. Studying preventive behavior also makes it possible to adapt recommendations in terms of preventive measures for cancer patients in the event of a new epidemic.

For example, a decline in preventive behaviors among cancer patients could be expected following vaccination. To increase preventive behavior in a population with a low level of preventive behavior, it will be useful for emphasizing the adoption of masks and frequent hand washing. On the other hand, where these behaviors have already been adopted, recommendations could be to reduce the number of outings and increase physical distancing. In terms of preventive measures, the target population would be male patients and patients living with children. Future analyses of the PAPESCO-19 study will focus on the preventive behavior of healthcare workers.

### Conclusion

Our results show that the cancer patient population was a homogeneous population in terms of preventive behavior against COVID-19. Surprisingly, no patient clinical characteristics were associated with preventive behavior.
