# Supplementary material for: Preventive Behaviors of French Cancer Patients and How They Changed During the COVID-19 Outbreak (PAPESCO-19 Study)
Source: Int J Public Health. 2025 Dec 2;70:1608450. doi: 10.3389/ijph.2025.1608450 (PMC12705467; doi:10.3389/ijph.2025.1608450)
Supplement: Supplementary file 1 [file Supplementaryfile1.docx]

**Electronic supplementary material**

1. Statistical analysis
2. Results

*Figure B1. Flow-diagram of study participants*

*Figure B2. Variable selection table*

*Figure B3. Graph of cancer patient responses to questionnaires by visit and by center*

*Table B1. Completers (including deceased) vs Drop-outs*

# Statistical analysis

## The preventive behavior latent process

Preventive behavior was considered a continuous process that could vary over time. The variables previously classified as defining the preventive behavior were assumed to be observed indicators reflecting the level of the unobservable (i.e. latent) variable of preventive behavior. A latent process approach was favored over the use of a sum score of responses to the observed definition variables. Thus, individual information from definition variables was fully exploited without aggregation and without assuming that each variable had the same importance in the definition of preventive behavior.

As our observed variables were binary or categorical items, a graded-response model (GRM) (9,10), a cumulative probit model from item response theory, was used to link the latent variable to the observed variables. In this model, preventive behavior, assumed to be a continuous and normally distributed variable, was characterized by its distribution parameters (mean and variance). A second set of parameters (discrimination and location) was also estimated, pertaining to the characteristics of the observed variables. The discrimination parameter for each variable defining the preventive behavior indicates the strength of the relationship between this variable and the latent variable for preventive behavior. A higher value for the discrimination parameter indicates that the associated variable was more important for defining preventive behavior. As for location parameters, they indicate the values of the thresholds of change in the successive answer categories for each observed variable. Therefore, they locate the thresholds of definition variables along the latent variable continuum for preventive behavior. The parameters for the GRM (distribution of the latent variable, discrimination and location parameters) were estimated using marginal maximum likelihood. To improve model convergence, the observed variables were recoded so that the reference answer category for each variable represents a high level of preventive behavior. This means that the higher the latent variable, the less preventive the behavior.

## Heterogeneity of the trajectories

Latent class mixed models were combined with the GRM to identify different profiles of preventive behavior. Latent class analysis was used to define subpopulations of patients according to their preventive behavior trajectory. Latent class mixed models are an extension of linear mixed models suited to heterogeneous subpopulation analysis. Rather than modelling a unique mean trajectory of preventive behavior along with individual deviations from this mean trajectory, a fixed number of latent classes was defined by a latent discrete variable. This latter variable was a grouping variable that was not directly observable but whose effects could be measured or observed through observed variables (11). Each patient belonged to a unique latent class. Latent class analysis did not directly assign individuals to latent classes during estimation, but rather the probabilities of belonging to each class were generated for each individual using a multinomial logistic model. The specific trajectory for preventive behavior in each class was defined using a linear mixed model with the latent process defined above as a dependent variable.

## Modeling strategy

The shape of the mean trajectory of the latent process of preventive behavior over time was determined by comparing BIC (with a lower value indicating a better fitting model) of five one-class latent linear mixed models, with different shapes: polynomials (quadratic, cubic) or quantile splines (3, 4 or 5 knots). As the grouping variable was latent, the number of latent classes was not known prior to analysis. Latent class mixed models were thus estimated using maximum likelihood for a number of latent classes varying from 1 to 5 to highlight some overall differences in the changes in preventive behavior in CPs and avoid pointing out a very specific subgroup. All classes had the same trajectory shape. Choosing the optimal number of classes was guided by the BIC, the size of the minimal latent class (10% or 15% depending on the number of classes) and the interpretability of the latent classes. Each model estimation was repeated from different sets of initial values to avoid potential convergence toward local suboptimal maxima.

After estimation, posterior latent class membership probabilities given the subjects response profiles were computed to classify each CP in the most likely class (modal class assignment). Posterior probabilities, as well as entropy, were inspected to assess the classification quality of the optimal model. The shape of the mean trajectory in each class was simplified by testing the related parameters to obtain the best fitting model.

To interpret the final best fitting model, the mean predicted trajectories of preventive behavior in each class and the location parameters were plotted.

In case of a single-class optimal model, the latent class mixed model was simply re-estimated with adjustment for center, sex, age and cancer location. Either a set of sociodemographic variables or a set of clinical variables, living conditions and self-reported COVID-19 related information was investigated in a backward selection procedure to identify the factors significantly (p < 0.10) associated with the preventive behavior.

# Results

Figure B1. Flow-diagram of study participants


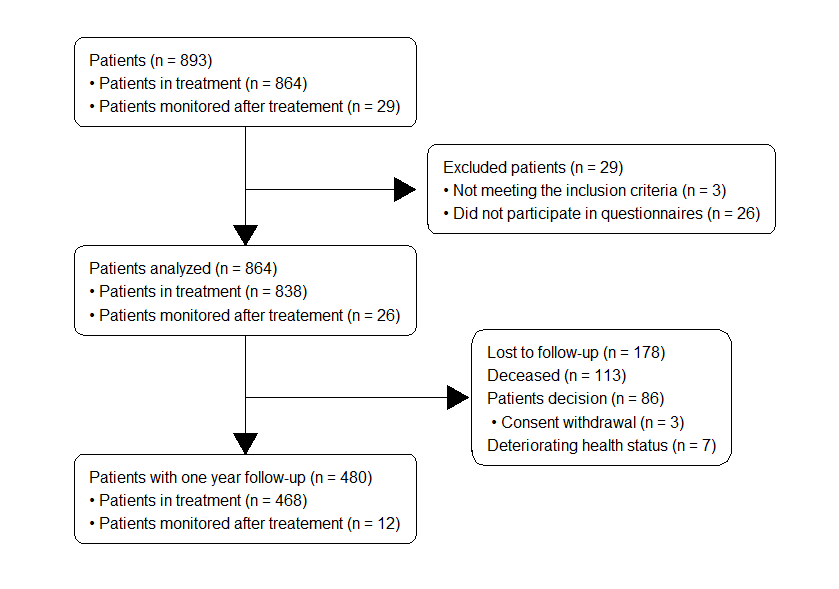


Figure B2. Variable selection table

| **Define preventive behavior** | **Influence preventive behavior** | **Not included** |
| --- | --- | --- |
| Lifestyle variables:  Wearing a mask  Hand washing  Number of times out of the home in the last 7 days  Physical distance | Socio-demographic variables:  Center  Sex  Age (Years)  Employment situation  Spend most of the day at home  House located  Lives with at least one other person  Children or grandchildren under 18 years old and number  Clinical variables:  Patients in treatment or in follow-up  Smoking status  Has at least one comorbidity  Location of primary tumor  Disease stage  Body mass index  Clinical variables related to COVID-19:  COVID-19 infection  Has been vaccinated | Clinical variables:  Current treatment at time of inclusion  Pre-inclusion treatment  Immunotherapy  Chemotherapy  Radiation therapy  Surgery  Hormone therapy  Targeted therapy  Percutaneous tumor ablation  Other  Lifestyle variables:  Means of transport to work  Walk, bike, scooter, etc.  Car, motorcycle, scooter  Cab, carpool, ambulance  Public transport  Did not go to work  Retired or on sick leave  Other means of transport  Means of transport for other trips  Walk, bike, scooter, etc.  Car, motorcycle, scooter  Cab, carpool, ambulance  Public transport  Other means of transport  Socio-demographic variables:  Job in contact with the public  Is working  Work on site  Full telework because of COVID  Full telework without COVID case  Partial telework because of COVID  Partial telework without COVID case  Sick leave because COVID  Sick leave without COVID case  Sabbatical leave  Technical or partial unemployment  Student, high school student, trainee  At home or on site  Looking for a job  Retired  Not working for health reasons (disability, chronic illness, …)  Without professional activity  Lives in his usual home  Size of the dwelling  Number of living rooms in the house  Presence of an outside access (balcony, garden, yard, …)  Co-ownership (building, residence) with common areas  Number of people living in this home  Spouse, partner  Children or grandchildren under 18 years old  Children or grandchildren over 18 years old  Parents  Other family members  Other people (friends, lodgers, …)  Number of children or grandchildren under the age of 18  Clinical variables related to COVID-19:  Intention to be vaccinated |

Figure B3. Graph of cancer patient responses to questionnaires by visit and by center


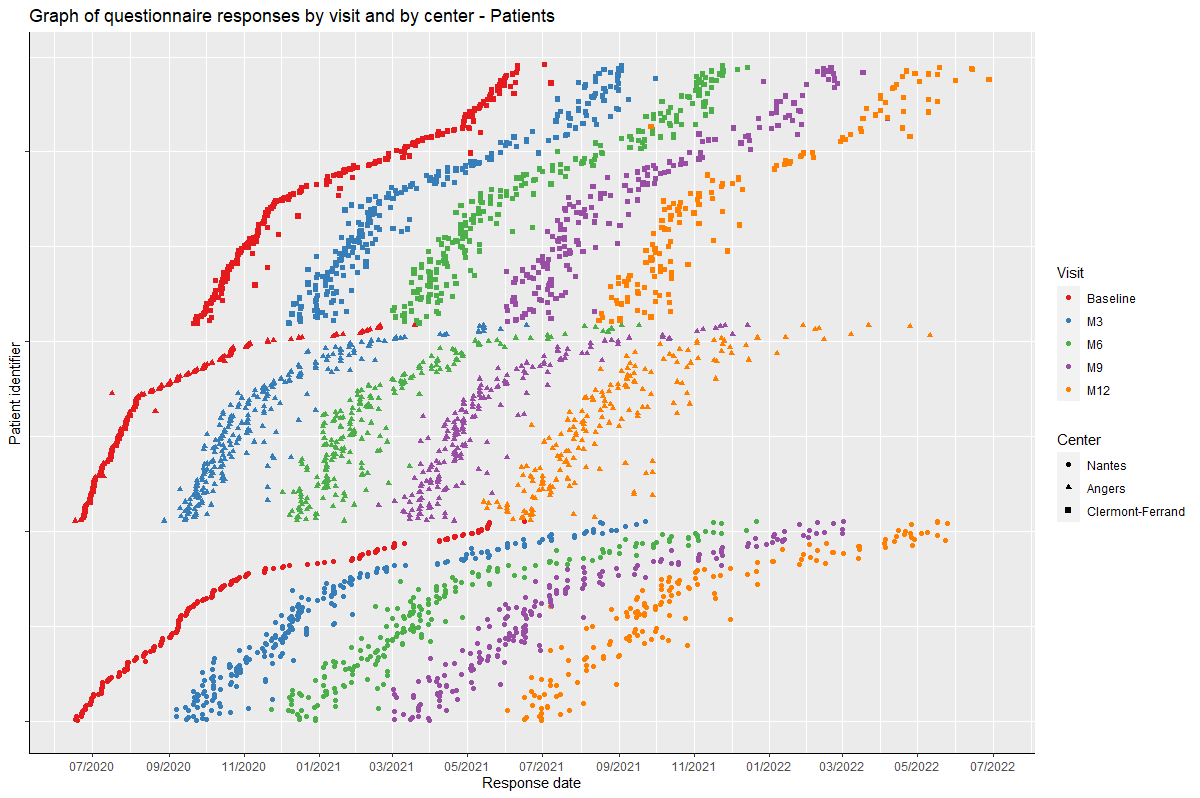


Clermont-Ferrand

Nantes

Angers

Second lockdown

Third lockdown

Table B1. Completers (including deceased) vs Drop-outs

| **Characteristic** | **Patients dropouts**  N = 271 | **Patients with one year follow-up or deceased**  N = 593 | **p-value**^1^ | |
| --- | --- | --- | --- | --- |
| **Center, n (%)** |  |  | <0.001 | |
| Angers | 51 (19%) | 208 (35%) |  | |
| Clermont-Ferrand | 150 (55%) | 192 (32%) |  | |
| Nantes | 70 (26%) | 193 (33%) |  | |
| **Sex, n (%)** |  |  | 0.5 | |
| Man | 91 (34%) | 186 (31%) |  | |
| Woman | 180 (66%) | 407 (69%) |  | |
| **Age, n (%)** |  |  | 0.6 | |
| 65 years and older | 124 (46%) | 260 (44%) |  | |
| Under 65 years old | 147 (54%) | 333 (56%) |  | |
| **Treatment status, n (%)** |  |  | 0.012 | |
| Patients in follow-up | 14 (5%) | 12 (2%) |  | |
| Patients in treatment | 257 (95%) | 581 (98%) |  | |
| **Cancer location, n (%)** |  |  | 0.020 | |
| Breast | 130 (49%) | 247 (42%) |  | |
| Digestive | 14 (5%) | 43 (7%) |  | |
| Lung | 13 (5%) | 56 (10%) |  | |
| Other | 50 (19%) | 80 (14%) |  | |
| Prostate | 18 (7%) | 38 (6%) |  | |
| Urological | 21 (8%) | 45 (8%) |  | |
| Uterine, cervical or endometrial | 22 (8%) | 78 (13%) |  | |
| Missing | 3 | 6 |  | |
| **Cancer stage, n (%)** |  |  | <0.001 | |
| Locally advanced | 45 (18%) | 117 (20%) |  | |
| Located | 107 (42%) | 132 (23%) |  | |
| Metastatic | 105 (41%) | 332 (57%) |  | |
| Missing | 14 | 12 |  | |
| **One or more comorbidities, n (%)** | 178 (66%) | 376 (63%) | 0.5 | |
| Missing | 1 | 0 |  | |
| **Tobacco smoking status, n (%)** |  |  | 0.4 | |
| Current smoker | 26 (10%) | 74 (12%) |  | |
| Former smoker | 82 (30%) | 163 (27%) |  | |
| Non-smoker | 163 (60%) | 356 (60%) |  | |
| **Body mass index classifications, n (%)** |  |  | 0.9 | |
| Normal weight | 111 (48%) | 242 (45%) |  | |
| Obesity | 35 (15%) | 90 (17%) |  | |
| Overweight | 75 (32%) | 181 (33%) |  | |
| Underweight | 11 (5%) | 29 (5%) |  | |
| Missing | 39 | 51 |  | |
| **Socio-professional category, n (%)** |  |  | 0.003 | |
| Craftsmen, merchants, company managers | 20 (8%) | 42 (8%) |  | |
| Employees | 58 (24%) | 143 (26%) |  | |
| Executives and higher intellectual professions | 34 (14%) | 94 (17%) |  | |
| Farmers | 13 (5%) | 7 (1%) |  | |
| Intermediate professions | 53 (22%) | 144 (27%) |  | |
| Never worked | 7 (3%) | 6 (1%) |  | |
| Other | 33 (14%) | 45 (8%) |  | |
| Workers | 22 (9%) | 60 (11%) |  | |
| Missing | 31 | 52 |  | |
| **Employment status, n (%)** |  |  | 0.3 | |
| Retired or without professional activity | 148 (60%) | 302 (54%) |  | |
| Sick leave or not working for health reasons | 75 (30%) | 190 (34%) |  | |
| Working or studying | 24 (10%) | 64 (12%) |  | |
| Missing | 24 | 37 |  | |
| **Spending most of the day at home, n (%)** |  |  | 0.052 | |
| No, must go out to work | 18 (7%) | 19 (3%) |  | |
| No, unrestricted | 34 (14%) | 77 (14%) |  | |
| Yes | 193 (79%) | 457 (83%) |  | |
| Missing | 26 | 40 |  | |
| **House location, n (%)** |  |  | 0.5 | |
| In a city of 20,000 to 100,000 residents | 34 (14%) | 62 (11%) |  | |
| In a city with a population of over 100,000 | 31 (13%) | 88 (16%) |  | |
| In a city with less than 20,000 residents | 60 (24%) | 133 (24%) |  | |
| In a rural area | 121 (49%) | 280 (50%) |  | |
| Missing | 25 | 30 |  | |
| **Living with at least one other person, n (%)** | 202 (78%) | 462 (80%) | 0.6 | |
| Missing | 12 | 12 |  | |
| **Number of children or grandchildren under the age of 18 at home, n (%)** |  |  | 0.12 | |
| No children | 214 (84%) | 473 (82%) |  | |
| One child | 14 (5%) | 54 (9%) |  | |
| Two children or more | 27 (11%) | 49 (9%) |  | |
| Missing | 16 | 17 |  | |
| ^1^Pearson's Chi-squared test; Fisher's exact test | | | |  |
